# MicroRNAs in cancer metastasis: biological and therapeutic implications

**DOI:** 10.1017/erm.2023.7

**Published:** 2023-03-17

**Authors:** Marie C. Sell, Charmaine A. Ramlogan-Steel, Jason C. Steel, Bijay P. Dhungel

**Affiliations:** 1 School of Health, Medical and Applied Sciences, Central Queensland University, Rockhampton, QLD 4701, Australia; 2 Gene & Stem Cell Therapy Program Centenary Institute, The University of Sydney, Camperdown, NSW 2050, Australia; 3 Faculty of Medicine & Health, The University of Sydney, Camperdown, NSW 2050, Australia

**Keywords:** Cancer therapy, cancer, metastasis, microRNA

## Abstract

Cancer metastasis is the primary cause of cancer-related deaths. The seeding of primary tumours at a secondary site is a highly inefficient process requiring substantial alterations in the genetic architecture of cancer cells. These alterations include significant changes in global gene expression patterns. MicroRNAs are small, non-protein coding RNAs which play a central role in regulating gene expression. Here, we focus on microRNA determinants of cancer metastasis and examine microRNA dysregulation in metastatic cancer cells. We dissect the metastatic process in a step-wise manner and summarise the involvement of microRNAs at each step. We also discuss the advantages and limitations of different microRNA-based strategies that have been used to target metastasis in pre-clinical models. Finally, we highlight current clinical trials that use microRNA-based therapies to target advanced or metastatic tumours.

## Introduction

**Fig. 1.**
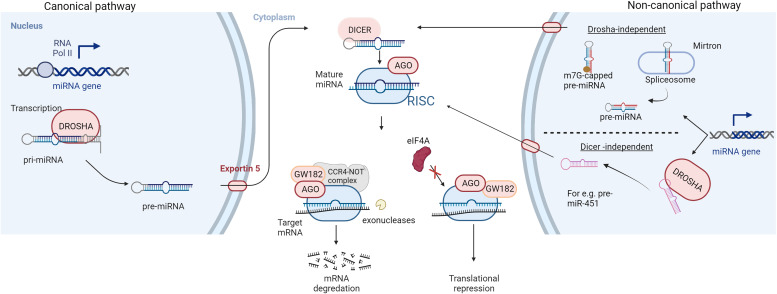
Biogenesis and mechanism of action of miRNAs: RNA polymerase II-mediated transcription forms the primary miRNA (pri-miRNA) which is cleaved by an RNase III enzyme (DROSHA) to produce a precursor miRNA (pre-miRNA) in the canonical pathway of miRNA biogenesis. The pre-miRNA is exported to the cytoplasm via exportin 5, for further processing by RNase III DICER to form a mature miRNA duplex. Non-canonical pathways are independent of Drosha or Dicer processing. The miRNA duplex is then unwound whereby the guide strand along with Argonaute (AGO) proteins form a miRNA-induced silencing complex (RISC). The RISC complex binds to target sequences of mRNA leading to translation repression or degradation. AGO recruits GW182 which forms a complex with CCR4-NOT making the target mRNA susceptible to cleavage by exonucleases while hindrance to the binding of eukaryotic initiation factor-4A (eIF4A) to the target mRNA leads to translational inhibition.

In 1993, a revolutionary discovery of the first microRNA (miRNA) while studying nematode Caenorhabditis elegans was made by Ambros *et al*. (Ref. 1). This finding revealed an essential part of the non-coding genome that plays a key role in post-transcriptional gene regulation (Ref. 1). Fast track 29 years, now over 2600 mature human miRNA sequences have been identified (Ref. 2). MicroRNAs are small, endogenous, single-stranded, non-protein coding RNA molecules of 19 to 24 nucleotides. MiRNAs account for approximately 3% of the human genome and are evolutionary conserved across mammals (Refs 3, 4). The canonical miRNA biogenesis pathway (Fig. 1) starts with RNA polymerase II-mediated transcription of the primary miRNA gene (pri-miRNA). The pri-miRNA is characterised by a hairpin structure with a 5′ cap and polyadenylation site at the 3′ (Ref. 5). Drosha, an RNase III protein, then cleaves the pri-miRNA, releasing a precursor loop (pre-miRNA) of approximately 70–100 nucleotides (Ref. 6). Upon release, the pre-miRNA is exported to the cytoplasm via Exportin 5 (Ref. 7). In the cytoplasm, pre-miRNA is cleaved by an RNase III called Dicer, to produce a double-stranded mature miRNA of approximately 22 nucleotides in length (Ref. 8). The non-canonical pathways utilise different combination of proteins during the biogenesis steps. These pathways can be further classified as Drosha- and Dicer- independent pathways. For instance, miRNAs produced from introns of messenger RNAs or mirtrons can bypass Drosha-mediated processing (Ref. 9). 7-methylguanosine (m7G)-capped pre-miRNA have been identified which are nascent RNAs directly exported to the cytoplasm through exportin 1 without the need for Drosha (e.g. pre-miR-320) (Ref. 10). Another example is the biogenesis of miR-451 which is independent of Dicer processing but requires Drosha and Argonuate 2 (AGO2) protein (Ref. 11).

The double-stranded mature miRNA is unwound, and the opposite strand is degraded. The remaining strand, the mature or the guide strand, is the final single-stranded miRNA molecule which forms a complex with the AGO proteins called the RNA-induced silencing complex (RISC) (Ref. 3). There are four mammalian AGO proteins (AGO1–4) but only AGO2 is able to cleave the target mRNA complementary to the miRNA (Ref. 12). The RISC complex binds to target messenger RNAs (mRNAs) which possess sequences complementary with the miRNA (Ref. 13). The binding of the RISC complex to mRNAs is mediated by a 6–8 nucleotide long region within the miRNA called the seed sequence or miRNA binding site (Ref. 14). The resulting miRNA-mRNA duplex leads to an inhibition or in some cases enhancement, of translation (Ref. 14). The miRNA target sites are usually located at the 3′-untranslated region (3′-UTR) of mRNA and the binding of the RISC complex leads to gene silencing by translation repression and mRNA decay (Ref. 15). Mechanistically, the AGO protein recruits the GW182 which interacts with the polyadenylate-binding protein PABPC to induce mRNA deadenylation (Ref. 16). This promotes decapping of the mRNA and makes it susceptible to degradation by 5′-3′ exoribonucleases (Ref. 17). For translational repression, GW182 recruits carbon catabolite repressor protein 4 complexes which in turn recruit RNA helicases like DDX6 (Refs 18, 19). MicroRNA-mediated inhibition of mRNA translation initiation results from the interference with the eukaryotic initiation factors eIF4A-I and eIF4A-II (Ref. 15). Although exact molecular details remain to be uncovered, existing evidences suggest that the RISC complex dissociates initiation factors from target mRNAs inhibiting the assembly of the translation initiation complex (Refs 20, 21).

Although a perfect complementarity with mRNAs is optimal for the function of miRNAs, some miRNAs can regulate mRNAs with partial complementarity (Ref. 22). Consequently, miRNA-mediated regulation of gene expression affects almost every fundamental cellular process, such as development, differentiation, proliferation, metabolism and apoptosis (Ref. 23). Unsurprisingly, the dysregulation of miRNA profile also significantly correlates with the onset and progression of cancer (Ref. 24). The impact of individual miRNAs on cancer often differs between cancer types. A miRNA can be tumour suppressive, oncogenic or a regulator of metastasis (Ref. 25). This review will focus on the role of miRNA in cancer metastasis. We will investigate miRNA dysregulation in different stages of the metastatic process and examine molecular drivers affected. We will summarise pre-clinical studies that have successfully employed miRNA-based therapies for metastatic cancer and highlight their limitations. Finally, we will examine miRNA-based therapies for the treatment of advanced and metastatic cancer that are currently in clinical trials.

### Metastasis

**Fig. 2.**
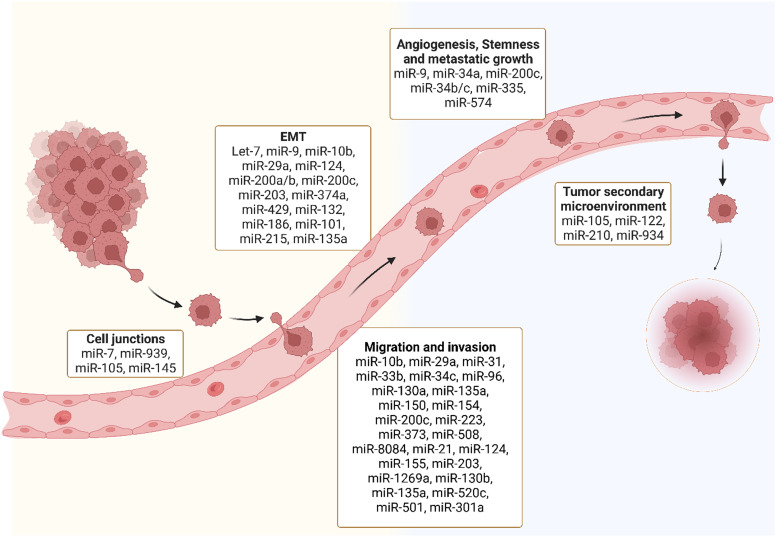
MiRNAs and different stages of metastasis: Several miRNAs are dysregulated throughout different stages of the metastatic process including disruption of tight junctions, epithelial to mesenchymal transition (EMT), migration and invasion, angiogenesis, stemness and metastatic growth and tumour secondary microenvironment.

The development of secondary tumours in distant organs, i.e. metastasis, is a hallmark of cancer (Ref. 26). The spread of cancer cells to secondary sites is the main cause of cancer-related morbidity and mortality (Ref. 27), yet we are only beginning to unravel the molecular mechanisms that drive metastasis (Ref. 28). Several phases are involved in the development of secondary tumours (Ref. 29). First, cancer cells loose adhesion factors and detach from the primary tumour allowing penetration and intravasation into the circulatory and lymphatic systems (Ref. 29). The cells in the vasculature called circulating tumour cells (CTCs), exploit mechanisms like cell cycle arrest, to evade and survive immune surveillance (Ref. 30). There is significant research aimed at studying and characterising CTCs which is beyond the scope of this review. Secondly, this process is followed by the extravasation and infiltration of the cells into distant capillary beds (Ref. 31). Finally, invasion and proliferation of the tumour in distant organs occurs (Ref. 31). Metastasis and the establishment of secondary tumours is a very inefficient process as the majority of tumour cells in circulation are eliminated (Ref. 32). The establishment of a microenvironment for cancer cells to seed, known as a premetastatic niche, is essential for the development of secondary cancer (Ref. 33) (Fig. 2). The most common locations of metastasis in the body are liver, bone, lung, nervous system, pleura and peritoneum (Ref. 34). In this review, we will focus on the role of miRNAs in metastasis. We will discuss different stages in the metastatic process and summarise miRNAs that have been reported to be involved in each of these steps.

## MicroRNA dysregulation in cancer metastasis

MiRNAs are regulators of virtually every cellular process, including the ones that lead to the development of metastasis (Ref. 35). MiRNA can target the mRNA of tumour suppressors or oncogenes implicated at different phases of the developing metastatic tumour (Table 1) (Ref. 36). Genetic mutations in cellular pathways resulting from the dysregulation of miRNA have been widely identified in the metastatic pathways (Ref. 37). Here we discuss miRNA dysregulation and how it impacts key stages of metastasis.

**Table 1.** A list of microRNAs involved at different stages of cancer metastasis

miRNA	miRNA expression	Pro/anti	Target genes/ effect	Primary tumour	Ref.
Cell Junction					
miR-105	High	Anti-cell junction	Targets *ZO-1* to destroy natural barrier	Breast	(Ref. 40)
miR-486-5p	High	Anti-cell junction	Targets *CADM1* to destroy natural barrier	NSCLC	(Ref. 41)
939-5p	High	Anti-cell junction	Downregulates CDH5/ enhance cell migration	Breast	(Ref. 42)
miR-145	Low	Pro-cell junction	Increased focal junction protein, paxillin increasing migration	CRC	(Ref. 43)
miR-7	Low	Pro-cell junction	increases FAK expression increasing migration	Colon	(Ref. 44)
Epithelial to mesenchymal transition					
Let-7	Low	Anti-EMT	Increase m-MYC, HMGA2 & KRAS expression	PDAC	(Ref. 57)
miR-9	High	Pro-EMT	Reduced *CDH1* downregulates E-cadherin	Breast	(Ref. 54)
miR-132	Low	Anti-EMT	Increase ZEB2, downregulation of E-cadherin, upregulation of vimentin & fibronectin.	CRC	(Ref. 50)
miR-132	Low	Anti-EMT	Increase ZEB2, downstream downregulation of E-cadherin & upregulation of vimentin.	NSCLC	(Ref. 53)
miR-186-5p	Low	Anti-EMT	Increased ZEB1 expression	CRC	(Ref. 52)
miR-200 family	Low	Anti-EMT	Increased ZEB1 & ZEB2 expression	CRC pancreatic lung breast gastric	(Refs 45–49)
miR-203	Low	Anti-EMT	Increased Annexin-A4 expression	Gastric	(Ref. 56)
miR-215	Low	Anti-EMT	Increased ZEB2 expression	NSCLC	(Ref. 51)
miR-374a	High	Pro-EMT	Suppress WIF1, PTEN, WNT5A, to activate Wnt/B-catenin signalling	Breast	(Ref. 55)
Migration and Invasion					
miR-10b	High	Pro-migration & invasion	Suppression of HOXD10	Breast Gastric CRC	(Refs 58-60)
miR-21-5p	High	Pro-migration & invasion	Suppression of PDCD4	Breast	(Ref. 63)
miR-29c	Low	Anti-migration & invasion	Upregulation of Integrin B1 & MMP2	Lung	(Ref. 66)
miR-96	High	Pro-migration & invasion	Inhibition of PTPN9	Breast	(Ref. 61)
miR-124	Low	Anti-migration & invasion	Increased ROCK1 expression	CRC	(Ref. 68)
miR-135a	High	Pro-migration & invasion	Suppression of HOXA10	Breast	(Ref. 62)
miR-150-5p	Low	Anti-migration & invasion	Increased HMGA2 expression	Breast	(Ref. 64)
miR-203	Low	Anti-migration & invasion	Increased EIF5A2 expression	CRC	(Ref. 67)
miR-373	Low	Anti-migration & invasion	Increased BRF2 expression	NSCLC	(Ref. 65)
miR-520c	High	pro migration & invasion	suppression of IRF2	gastric	(Ref. 69)
Angiogenesis, Stemness and Metastatic Growth					
miR-30c	High	Pro-stemness	Increased OCT4, SOX2, KLF4 and NANOG	Breast	(Ref. 70)
miR-129-2	Low	Pro-metastatic growth	Increased SOX4 expression	PDAC	(Ref. 71)
miR-145	Low	Anti-angiogenesis	Increased N-RAS and VEGF signalling pathways	Uveal melanoma	(Ref. 72)
miR-200c	Low	Anti-stemness	Increased SOX2 & KLF4 stemness gene expression	Breast	(Ref. 70)
miR-335	Low	Pro-metastatic growth	Increased SOX4 expression	PDAC	(Ref. 71)
miR-574-5p	High	Pro-angiogenesis	Suppression of PTPRU/ enhanced tyrosine phosphorylation of *β*-catenin	NSCLC	(Ref. 73)
Tumour Microenvironment					
miR-301a-3p	High	Pro-tumour microenvironment	Mediates M2 macrophage via PTEN/PI3Kℽ	Pancreatic	(Ref. 74)
miR-130b-3p	High	Pro-tumour microenvironment	Mediates M2 macrophages and cancer cells communication	Gastric	(Ref. 75)
miR-934	High	Pro-tumour microenvironment	Downregulation of PTEN and activation of PI3K/AKT signalling pathway	CRC	(Ref. 76)
miR-148b	Low	Anti-tumour microenvironment	Increased CSF1 enhancing TAM infiltration	HCC	(Ref. 77)
miR-501-3p	High	Pro-tumour microenvironment	TGFBR3 inhibition; activating TGF-B signalling pathway	PDAC	(Ref. 78)
miR-214	High	Pro-tumour microenvironment	Induction of Tregs secreted IL-10	Breast HCC NSCLC Pancreatic	(Ref. 79)

### MiRNAs and cell junctions

MiRNAs regulate the transcription of cellular junction proteins critical for signalling communication, growth and migration (Ref. 38). For metastasis to occur, the disruption of cellular junctions is essential (Ref. 39). Several miRNAs regulate the expression of zonula occluden-1 (*ZO-1*), a major component of tight junctions (Ref. 40), e.g. miR-105 regulates the metastasis of breast cancer by inhibiting ZO-1 (Ref. 40). Furthermore, overexpression of miR-105 induces the metastasis of cancer cells to distant organs including the liver (Ref. 40). The cell-cell adhesion for the adaption of the premetastatic niche is promoted by oncogenic miR-105 through the tight junction protein *ZO-1* (Ref. 40).

A highly elevated expression of miR-486-5p in CD31^+^ vascular endothelial (VE) cells increases permeability and promotes non-small cell lung cancer (NSCLC) metastasis (Ref. 41). The transfection of human VE cells, with miR-486-5p antagomirs, targets *CADM1* and destroys the tight junctions of VE cells. Similarly, the downregulation of adherens junction protein, VE cadherin (CDH5), by miRNA, enhances breast cancer cell migration (Ref. 42). MDA-MB-231-GFP breast cancer cells transfected with miR-939 mimics, show higher migration through the endothelial barrier which is mediated by a downregulation of CDH5 (Ref. 42). MiR-145 negatively correlates with the focal junction protein paxillin in colorectal cancer (CRC) (Ref. 43). Delivery of miR-145 mimics downregulates paxillin and inhibits cell proliferation, migration and invasion (Ref. 43). Similarly, miR-7 targets focal adhesion kinase (FAK) expression to suppress colon cancer proliferation, migration and invasion (Ref. 44). HCT-8 and Caco-2 colon cancer cell lines transfected with miR-7 mimics have been shown to have a negative correlation with colon cancer metastasis (Ref. 44).

### MiRNAs in epithelial to mesenchymal transition (EMT)

Epithelial to mesenchymal transition (EMT) of cells is mostly controlled by zinc finger E-box binding protein transcription factors (TFs) *ZEB1* and *ZEB2* and leads to an upregulation of vimentin and N-cadherin and repression of E-cadherin (Ref. 45). The miR-200 family (miR-200a, miR-200b, miR-200c, miR-124, miR-429) has been extensively investigated as a regulator of *ZEB1* and *ZEB2* in cancer that mostly metastasises to the liver, including colorectal (Ref. 45), pancreatic (Ref. 46), lung (Ref. 47), breast (Ref. 48) and gastric cancers (Ref. 49). *ZEB1* and *ZEB2* are direct targets of several miRNAs in CRC and NSCLC. Zheng *et al*. reported a significant downregulation of miR-132 in CRC and furthermore used a luciferase activity assay to demonstrate miR-132-mediated regulation of *ZEB2* (Ref. 50). MiR-215-mediated regulation of *ZEB2* expression has been demonstrated in CRC while low levels of miR-186-5p in CRC inhibit EMT by targeting *ZEB1* (Refs 51, 52). Additionally, it was reported that miR-132 directly targets *ZEB2* in NSCLC driving EMT (Ref. 53). There are other miRNAs that promote EMT in breast cancer. MiR-9 repression of E-cadherin, increases EMT in breast cancer (Ref. 54) while oncogene miR374a targets negative regulators of Wnt/B-catenin pathway including *WIF1*, *PTEN* and *WNT5A* and activates the Wnt/B-catenin pathway to promote EMT in breast cancer (Ref. 55). The downregulation of miR-203 promotes EMT in gastric cancer by releasing the repression of its target gene Annexin A4 (Ref. 56). In pancreatic ductal adenocarcinoma (PDAC), EMT is driven by an increase in the expression of *c-MYC*, *HMGA2* and *KRAS* mediated by a reduction in Let-7 miRNA expression (Ref. 57).

### MiRNAs in cancer cell migration and invasion

MiR-10b was the first miRNA to be associated with metastasis in patients with advanced breast cancer (Ref. 58). MiR-10b promotes the migration and invasion in different cancer types including colorectal, breast and gastric cancers by regulating *HOXD10* (Refs 58–60). In breast cancer, migration and invasion is enhanced by an upregulation of miR-96, miR-135a and miR-21 and the downregulation of miR-150. Similarly, in breast cancer, oncogenic miR-96 targets *PTPN9* (Ref. 61) and miR-135a represses *HOXA10* to drive the migration and invasion (Ref. 62). Another oncogene miR-21-5p upregulates the expression of programmed cell death protein 4 (PDCD4) to increase breast cancer invasion and migration (Ref. 63). Tumour suppressor miR-150, which targets *HMGA2*, is aberrantly expressed in breast cancer and promotes migration and invasion (Ref. 64). In NSCLC, a downregulation of miR-373 which regulates TFIIB-related factor 2 (*BRF2*), promotes cell migration and invasion (Ref. 65). Multiple investigations of cancers metastasising to the liver including pancreatic cancer and lung cancer, demonstrate that cell migration and invasion is controlled by miR-29c-mediated regulation of *MMP2* (Ref. 66). Low levels of miR-124 and miR-203 promote migration and invasion in CRC by targeting *ROCK1* and eukaryotic initiation factor 5A2, respectively (Refs 67, 68). Similarly, gastric cancer migration and invasion is driven by the dysregulation in miR-520c-mediated regulation of *IRF2* (Ref. 69).

### MiRNAs in stemness, angiogenesis and metastatic growth

Key events of metastatic growth and tumour mediated angiogenesis are regulated by miRNAs. Downregulation of miR-200c and overexpression of miR-30c targets stemness-related genes in breast cancer influencing secondary tumour growth (Ref. 70). Oncogene miR-9-mediated repression of E-Cadherin contributes to an overexpression of vascular endothelial growth factor (VEGF) leading to an increase in angiogenesis which is essential for the growth of secondary tumour (Ref. 54). The co-repression of miR-129-2 and miR-335 significantly upregulates oncogenic *SOX4* driving metastatic growth in PDAC (Ref. 71). Likewise, VEGF overexpression leading to angiogenesis, tumour growth and invasion in uveal melanoma was significantly suppressed by miR-145 mimics directly targeting N-RAS and VEGF signalling pathways (Ref. 72). Upregulation of miR-574-5p promotes metastatic growth in NSCLC enhancing tyrosine phosphorylation of B-catenin via the repression of protein tyrosine phosphate receptor type U (PTPRU) (Ref. 73).

### MiRNAs in tumour microenvironment

MiRNA is an important mediator of the crosstalk between the tumour microenvironment and tumour cells, playing an important role in metastasis progression. Tumour-associated macrophages (TAM) are key components of the tumour microenvironment, regulated by miRNAs, to exhibit pro-tumour activity in the microenvironment. For example, highly expressed miR-301a in pancreatic cancer cells induces M2 macrophage polarisation via the PTEN/PI13Kℽ signalling pathway to promote pancreatic cancer cell metastasis (Ref. 74). Similarly, miR-130-3p upregulated in gastric cancer mediates communication between M2 macrophages and cancer cells in the tumour microenvironment by promoting the expression of mixed lineage leukaemia 3 (*MLL3*) gene and grainyhead-like 2 (*GRHL2*) gene (Ref. 75). Tumour-derived exosomal miR-934 promotes liver metastasis of CRC by regulating the interaction between TAMs and the metastatic microenvironment (Ref. 76). Downregulated miR-148b expression negatively correlates with the upregulation of colony-stimulating factor-1 (CSF1), promoting CSF1 signalling and inducing TAM infiltration to promote hepatocellular carcinoma (HCC) metastasis (Ref. 77). Furthermore, M2 macrophage-derived exosomal miR-501-3p downregulated TGFRR3 to promote liver and lung metastasis of PDAC in nude mice by activating the TGF-*β* signalling pathway (Ref. 78). Upregulated miR-214 negatively correlates with PTEN in several cancers including breast, HCC, NSCLC and pancreatic cancer. The upregulated miR-214 promotes regulatory T-cells (Tregs) which secret high levels of IL-10 and enhance immune suppression for metastatic progression (Ref. 79).

## Regulation of metastatic miRNAs

The inter-regulation between miRNAs and different TFs lead to a finely tuned and spatio-temporally regulated transcriptional and post-transcriptional gene regulation system which gets perturbed during metastasis (Ref. 80). Evidences suggest that the dysregulation of miRNAs in cancer can occur at the genomic level. An example is the frequently lost genomic locus of miR-146a in acute myeloid leukaemia (Ref. 81). However, aberrations at the transcription level are widely studied and thought to be more impactful. For instance, tumour suppressive TF p53 regulates the expression of the miR-16, miR-145 and miR-34 family (Ref. 82). While miR-145 is repressed by the oncogenic RAS-responsive element-binding protein 1 (RREB1) (Ref. 83). Other reported TFs that regulate miR-145 include CCAAT/enhancer-binding protein beta, beta-catenin/T cell factor 4 and forkhead TFs FOXO1 and FOXO3 (Refs 84, 85). The oncogenic c-Myc TF suppresses expression of miRNAs 29, 30 and let-7 family (Refs 86–88). ZEB1 and ZEB2, key activators of EMT repress the expression of miR-200 family of genes (Ref. 89) including miR-200c (Ref. 90). Similarly, studies have demonstrated that nuclear receptors, especially estrogen receptor (ER) and androgen receptor (AR) can directly regulate the transcriptional activity of miRNAs in cancer by binding to promoter or repressor regions. For instance, ER binds to the promoter region of the miR-221 and inhibits its expression in breast cancer (Ref. 91). Interestingly in prostate cancer, a negative feedback loop that regulates miR-135a and AR protein expression in an androgen-dependent manner was identified. Here, androgen stimulates the expression of miR-135a which inhibits AR expression. In turn, AR binds to the miR-135a locus and controls its expression (Ref. 92). Some studies have also indicated roles of epigenetic factors like DNA methylation in the regulation of metastatic miRNAs like miR-200c (Refs 93, 94). Similarly, the promoter of miR-34a is hypermethylated in ovarian cancer (Ref. 95). Other factors that modulate miRNA activity like regulation and post-translational modifications of AGO proteins (Ref. 96), miRNA transport to the cytoplasm and regulation of miRNA–mRNA interactions need to be further explored in the context of metastasis.

Several recent studies have also compared changes in miRNA expression in primary and secondary tumours to identify potential drivers of metastasis. For instance, a study involving 33 CRC patients with metastasis and 14 patients without metastasis revealed differential expression of 17 miRNAs and their 198 predicted targets. There was a strong association of the target genes with cancer progression and metastasis (Ref. 97). In another study involving metastatic breast cancer patients, the upregulation of miR-342-3p and miR-187-3p was associated with an increased progression-free survival (PFS) and overall survival (OS); while, the downregulation of miR-301a-3p was associated with a higher PFS and OS (Ref. 98). In addition to being therapeutic targets, studies in several different cancer types have identified differences in expression levels of several miRNA indicating that they may serve as diagnostic or prognostic markers (Refs 99–106).

## MiRNA-based therapies

Endogenous miRNAs play a crucial role in maintaining cellular homoeostasis (Ref. 107). The genomic and transcriptomic alterations in cancer cells can perturb the global miRNA expression profile causing genome-wide transcriptional changes (Ref. 108). These changes can lead to an upregulation of oncogenes and/or a downregulation of tumour suppressors which is critical for metastasis (Ref. 108). Most miRNA-targeted cancer therapies focus on restoration or inhibition of dysregulated miRNAs (Ref. 109) but recently, miRNA-based detargeting strategies have been utilised for cell/tissue-specific targeted therapies (Ref. 110). Table 2 provides a comprehensive list of miRNA-based therapeutic strategies which will be discussed in this section in detail.

**Table 2.** MiRNA-based therapies for cancer metastasis: Dysregulated miRNAs are potential therapeutic targets to treat metastatic cancer

MiRNA-based therapies	miRNA	Cancer	Therapy effects	Model	Ref.
MiRNA replacement					
miRNA mimics	miR-149-3p	Breast	Regulation of Foxp1 expression in CD8+ T-Cells	Homograft mouse model	82
	miR-140-5p	NSCLC	Higher adhesion, changes to EMT	A549 & SK-MES1 cell lines	83
	miR-195-5p miR-101-3p miR-338-5p	Lung	Combination delivery yields reduced tumour growth	Animal model	84
	miR-34a	Lung	combination radiation/mimic regulates double-strand break repair		85
MiRNA inhibition					
ASOs	miR-21	NSCLC	Regulation of PTEN	H1650 cell line	88
	miR-21	Colon	Reduced VEGF expression	HCT116 cell line	96
	miR-21 miR-10b	TNBC	Multiple oncogenic miRNAs targeted for cumulative therapeutic effects	Mouse model	97
Antagomir	miR-210	Pancreatic	Combined delivery of miR-210 and siRNA-KRAS reduces metastasis.	Mouse model	89
MiRNA Sponges	miR-10b	Breast	Upregulation of HOXD10 inhibiting cancer growth and proliferation, migration and invasion.	MDA-MB-231 and MCF7	99
	miR-155 miR-21 miR-221 miR-22	Breast Pancreatic	Multi-potent sponge inhibits multiple miRNA regulating Foxo3a, PTEN and RhoA	Cell lines	100
MiRNA Mask	miR-522	NSCLC	Protects DENN2D expression to reduce migration and invasion	Cell lines	91
LNA anti-miR	miR-21	CRC	Inhibits cell proliferation	LS174T	92
	miR-9	Gastric	Promotes CDH1 expression to re-establish E-cadherin expression	Cell lines	101
	miR-663a miR-4787-5p	Pancreatic	Reduced TGF*β*1-induced EMT	Orthotopic mouse model	102
Small RNA Zipper	miR-221	Breast	Inhibits miRNA to increase target genes	cell lines	93
MiRNA-mediated Detargeting					
miRNA Detargeting	miR-122 miR-7 miR-148a	Pancreatic	Successful detargeting of the liver, brain and GT tract	Cell lines and xenograft models	104
	miR-148a miR-216a	Metastatic PDAC Liver	Successful targeting for reduced cancer growth and metastasis		105
	miR-122a miR-199a	HCC	Prevented off-targeting effects by sparing normal liver cells.	Cell lines	106–108

### MiRNA replacement

**Fig. 3.**
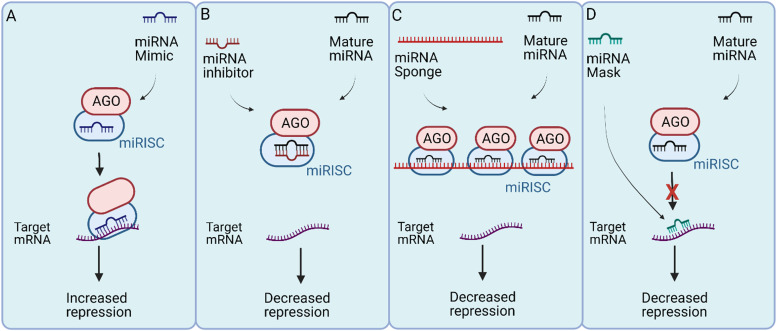
MiRNA-based therapies. (a) MiRNA replacement with mimics function like an overexpression of endogenous miRNA and increase the degradation or repression of target mRNAs. (b) The miRNA inhibitor approach minimises the binding of miRNA-induced silencing complex (miRISC) to target mRNAs. Different strategies used for miRNA inhibition includes antisense oligonucleotides (ASOs), antagomir antisense oligonucleotides, locked nucleic acid (LNA), antisense oligonucleotide and small RNA zippers. (c) MiRNA sponge binds to the miRISC complex reducing its binding to the target mRNA. (d) MiRNA mask prevents the miRISC from binding to the mRNA by ‘masking’ the miRNA binding site.

The restoration of miRNAs that are downregulated in cancer is one approach to target metastasis (Ref. 4). MiRNAs in metastatic cells can be restored using miRNA mimics (Ref. 111) which are small synthetic RNA duplexes containing an antisense strand with the same sequence as the endogenous miRNA (Ref. 112). To increase stability of the duplex and to enhance cellular uptake, the sense strand can be chemically modified. The sense strand may also contain several mismatches to minimise off-target effects (Ref. 112). Like the naturally occurring miRNA, these miRNA mimics are loaded into the RISC complex and inhibit downstream targets (Fig. 3a) (Ref. 112). MiRNA mimics have been widely studied for therapeutic purposes in both *in vitro* and *in vivo* cancer models (Ref. 113). For instance, miR-149-3p mimics suppress breast cancer growth and metastasis by regulating inhibitory receptors and *Foxp1* gene expression in CD8+ T cells in a homograft mouse model (Ref. 113). Treatment with miR-149-3p mimics reduced the apoptosis of CD8+ T cells which mediate the immune surveillance of cancer cells (Ref. 113). The promotion of CD8+ T cells resulted in the death of 4T1 mouse breast cancer cells (Ref. 114). Similarly, a reduction of migration and invasion in A549 and SK-MES1 squamous carcinoma NSCLC cell lines was observed after the transfection of miR-140-5p mimics (Ref. 114). The authors also reported a higher adhesion to an artificial extracellular matrix (ECM), indicating a change in EMT (Ref. 114). A combined delivery of three miRNA mimics, miR-195-5p, miR-101-3p and miR-338-5p, is more effective in reducing tumour growth and the number of metastatic nodules in animal models of lung cancer (Ref. 115). Similarly, delivering miR-34a mimic sensitises primary and metastatic derived lung cancer cell lines to radiotherapy, *in vitro* and *in vivo* (Ref. 116). Several other miRNA replacement therapies are currently being tested in both clinical and pre-clinical settings.

### MiRNA inhibition

Another approach to target metastasis is to inhibit upregulated oncogenic miRNAs (Ref. 117). The inhibition of oncogenic miRNAs overexpressed during metastasis can restore silenced tumour suppressors (Ref. 117). MiRNA inhibitors are single-stranded oligonucleotides complimentary to an endogenous miRNA (Ref. 4). These inhibitors can bind to endogenous miRNAs and inhibit their incorporation into the RISC complex (Fig. 3b) (Ref. 118). Several types of miRNA inhibitors have shown therapeutic advantages both *in vitro* and *in vivo* including antisense oligonucleotides (ASOs) (Ref. 119), antagomirs (Ref. 120), miRNA sponges (Ref. 121), miRNA masks (Ref. 122), locked nucleic acid (LNA) anti-miRNAs (Ref. 123) and small miRNA Zippers (Ref. 124).

#### Synthetic Antisense Oligonucleotides (ASOs)

ASOs are single-stranded, chemically modified DNA molecules, 20-25 nucleotides in length, with a full complementarity to a target miRNA (Ref. 125). ASOs inhibit the binding of mature miRNA to its target mRNA by producing an ASO-miRNA duplex which can lead to the cleavage of the miRNA and the upregulation of the target mRNA (Ref. 125). ASOs have already been approved by the Food and Drug Administration for the treatment of Duchenne muscular dystrophy and spinal muscular atrophy whereby exon skipping strategies are utilised to restore the dystrophin expression (Ref. 126). For cancer therapy and metastasis inhibition, some pre-clinical studies have been reported with ASOs. For example, Ge *et al*. designed an ASO to target miR-21 which is overexpressed in NSCLC. MiR-21 regulates the activity of PTEN, a regulator of invasion and a metastasis promoter (Ref. 119). The ASO-based drug was successful in reducing miR-21 expression and induced apoptosis in H1650 NSCLC cell line (Ref. 119). Likewise, the transfection of synthetic ASOs targeting miR-21 significantly reduced migration and invasion of HCT116 human colon carcinoma cell line accompanied by a reduction in the expression of VEGF which is critical for colon cancer metastasis (Ref. 127). Multiple oncogenic miRNAs can also be targeted simultaneously for additive therapeutic effects (Ref. 128). ASOs targeting miR-21 and miR-10b have been successfully delivered in cell lines and tumour xenografts for triple-negative breast cancer (TNBC) (Ref. 128). The simultaneous delivery of these miRNAs induces cancer apoptosis and inhibits tumour growth and metastasis in a mouse model of TNBC (Ref. 128).

#### Antagomir antisense oligonucleotides

Antagomirs are artificially synthesised single stranded RNA of 23 nucleotides length complementary to a miRNA. Antagomirs can be chemically modified with a cholesterol moiety for greater stability (Ref. 120). In a mouse model of pancreatic cancer, a cholesterol-modified polymetric CXCR4 antagonist was delivered with nanoparticles via an intraperitoneal delivery to localise efficacy and limit systemic side (Ref. 120). The co-delivery of antagomirs against miR-210 and siRNA against KRAS to this model demonstrated a reduced metastatic activity (Ref. 120). Of particular importance was the complete inhibition of liver metastasis, the primary metastatic site of PDAC (Ref. 120).

#### MiRNA sponges antisense oligonucleotide

MiRNA sponges are short, synthetic transcripts with the same sequence as the 3′UTR of mRNAs targeted by the miRNA. Acting as a decoy, sponges inhibit the ability of miRNAs to regulate their target mRNAs (Fig. 3c) (Ref. 121). There are some reports where miRNA sponging has been successfully performed for a single miRNA (Ref. 129). Liang, Zhang, Zhou, Wu, Lin and Liu (Ref. 130) designed a miRNA sponge plasmid to target miR-10b in metastatic breast cancer cell lines, MDA-MB-231 and MCF-7, demonstrating an inhibition of miR-10b and upregulation of its target *HOXD10*. This resulted in an inhibition of cancer growth and proliferation as well as a reduction in migration and invasion (Ref. 130). Additionally, a multi-potent miRNA sponge that simultaneously inhibits 4 oncogenic miRNAs, miR-155, miR-21, miR-221 and miR-222 was developed (Ref. 131). This multi-potent miRNA sponge was successful in inhibiting multiple oncogenic miRNAs, thus promoting anti-tumour effects in human breast cancer and pancreatic cancer cells (Ref. 131). Results demonstrated the multi-potent miRNA sponge to be more effective in inhibiting proliferation when compared to single miRNA-targeted sponges and demonstrated a 1.3-2.3-fold change in the protein levels of Foxo3a, PTEN and RhoA which are associated with an increased metastatic potential (Ref. 131).

#### MiRNA-masking antisense oligonucleotide

MiRNA-Masking (miR-Mask) is an inverted approach to protect mRNAs from miRNA-mediated repression (Ref. 122). In this approach, the miR-Masking oligonucleotides shield the miRNA binding sites of the mRNA to be protected (Fig. 3d) (Ref. 122). A full complementarity is required for better specificity (Ref. 122). This approach inhibits miRNA-mediated repression of targeted mRNAs without effecting the expression and potentially important functions of a miRNA (Ref. 122). Zhang *et al*. studied the effects of a miR-mask designed to complement the miR-522 binding site within *DENND2D* for the treatment of NSCLC and observed a reduced cell migration and invasion in NSCLC cells (Ref. 122).

#### Locked nucleic acid (LNA) antisense oligonucleotide

Another alternative oligonucleotide designed to inhibit miRNA oncogenic function are locked nucleic acid anti-miRs (LNA-i-miR) (Ref. 123). LNAs are chemically modified by connecting the 2′ oxygen and 4′ carbon to form an extra methylene bridge locking the ribose ring (Ref. 123). This leads to a higher thermal and *in vivo* stability and a greater binding affinity with mRNA targets (Ref. 123). LNA against miR-21 was effective in reducing the invasiveness and inhibited the proliferation of human colorectal adenocarcinoma cells (Ref. 123). Likewise, Lima *et al*. devised a strategy whereby LNA was efficient even when delivered at a low dose. In their study, miR-9 was targeted with LNAs to promote the expression of *CDH1* for the reestablishment of E-cadherin in human gastric cancer cells (Ref. 132). In another study, the delivery of LNA-i-miRs against miR-663a and miR-4787-5p reduced tumour burden and metastasis in an orthotopic mouse model of pancreatic cancer by decreasing TGF*β*1-induced EMT (Ref. 133).

#### Small RNA zippers

In this approach, oligonucleotides complementary to the second and the first half of a miRNA are synthesised and delivered into the cells (Ref. 124). Small RNA zippers connect multiple copies of a miRNA end-to-end by forming a duplex of multiple miRNA copies and inhibit the function of the target miRNA (Ref. 124). Like chemically modified LNAs, small RNA zippers have increased affinity, specificity and stability (Ref. 124). A 70–90% inhibition of miR-221 and miR-17 and rescue of their target genes was observed in breast cancer cell lines using miRNA zippers (Ref. 124). Further, the oncogenic effects of miR-221 were reversed by miR-221 zippers as demonstrated by the cell migration assay. However, the *in vivo* applications of miRNA zippers are yet to be tested (Ref. 124).

### MiRNA-mediated detargeting

Unlike miRNA replacement or inhibition therapies, this approach utilises the binding sites of miRNAs that are downregulated in cancer for detargeting the therapy from the normal cells and thereby reduce off-target effects. This approach is mostly useful for genetic therapies which utilise therapeutic gene transfer (Ref. 134). For instance, Baertsch *et al*. utilised three miRNAs; miR-122, miR-7, miR-148a, expressed at high levels in the liver, brain and the gastrointestinal tract, respectively, and demonstrated successful detargeting of these organs for a measles virus-mediated oncolytic virotherapy of pancreatic cancer in cell lines and murine xenograft models (Ref. 135). In another example, the binding sites of miRNAs downregulated in PDAC, miR-148a and miR-216a, were used for detargeting in locally advanced and metastatic pancreatic and liver cancer (Ref. 136). 8-miR148aT demonstrated detargeting effects by repressing all miR-148/152 family members in the pancreas and liver (Ref. 136). This study demonstrated that this method was highly efficient for targeted therapies as a significant decrease in cancer growth and metastasis was observed (Ref. 136). To prevent off-targets effects in the liver after suicide gene therapy, the binding sites of miR-122a and miR-199a, which are significantly downregulated in HCC, were used in multiple studies. Adeno-associated virus-based vectors were used to deliver miRNA122a and/or miRNA199a-regulated the suicide gene therapy system cytosine deaminase (CD)/ 5-fluorocytosine (Refs 137–139). Limited killing of normal liver cells with this system demonstrated an efficient liver detargeting using the binding sites of these miRNAs (Refs 137–139).

## MiRNA-based therapies in clinical trials

MiRNA-based strategies have demonstrated therapeutic potential in a range of conditions including advanced cancers (Ref. 140). In fact, several miRNA-targeted therapeutics are at different phases of clinical development for the treatment of advanced cancers and metastases (Ref. 141). For cancer therapy, miRNA therapeutics are injected directly into the site of the tumour which can increase the specificity, efficacy and reduce off-target effects (Ref. 112). Below we summarise clinically applied miRNA-targeted therapies (Table 3). Although not exclusive to metastasis, these therapies have shown promise in treating advanced cancers including those with metastasis to secondary organs.

**Table 3.** A list of cancer therapy clinical trials utilising miRNA-based strategies

MiR/Clinical trial identifier	Study	Condition	Drug	Phase
miR-34 NCT01829971	A Multicenter Phase I Study of MRX34, MicroRNA miR-RX34 Liposomal Injection	Primary Liver Cancer, SCLC Lymphoma Melanoma, Multiple Myeloma, Renal Cell Carcinoma, NSCLC	MRX34	Phase 1
miR-16 NCT02369198	MesomiR 1: A Phase I Study of TargomiRs as 2nd or 3rd Line Treatment for Patients with Recurrent MPM and NSCLC	Malignant Pleural Mesothelioma, Non-Small Cell Lung Cancer	TargomiRs	Phase 1
miR-155 NCT03603431	Safety, Tolerability and Pharmacokinetics of MRG-106 in Patients with Mycosis Fungoides (MF), CLL, DLBCL or ATLL	Cutaneous T-cell Lymphoma (CTCL), Mycosis Fungoides (MF), Chronic Lymphocytic Leukaemia (CLL)	Cobomarsen	Phase 1
miR-155 NCT03713320	SOLAR: Efficacy and Safety of Cobomarsen (MRG-106) vs. Active Comparator in Subjects with Mycosis Fungoides	Cutaneous T-Cell Lymphoma/Mycosis Fungoides	Cobomarsen Vorinostat	Phase 2
miR-155 NCT03837457	PRISM: Efficacy and Safety of Cobomarsen (MRG-106) in Subjects with Mycosis Fungoides Who Have Completed the SOLAR Study (PRISM)	Cutaneous T-Cell Lymphoma/Mycosis Fungoides	Cobomarsen	Phase 2
miR-193a-3p NCT04675996	First-in-Human Study of INT-1B3 in Patients with Advanced Solid Tumours	Advanced Solid Tumour	INT-1B3	Phase 1
miR-221 NCT04811898	A Dose Escalation Study of LNA-i-Mir-221 for Cancer Treatment	Multiple Myeloma, Refractory Hepatocarcinoma Advanced Solid Tumour	LNA-i-miR-221	Phase 1

### Clinical miRNA replacement

Tumour suppressor miR-15/16 family is downregulated in various cancers including lung and colon cancer that metastasise to the liver (Ref. 142). Downregulation of miR15/16 can increase drivers of metastasis including tumour growth, angiogenesis, EMT and stemness (Ref. 141). In pre-clinical studies, miR-16 mimic replacement safely inhibited growth and metastasis of Malignant Pleural Mesothelioma (MPM) and NSCLC xenograft tumours (Ref. 143). TargomiRs are minicells coated with an anti-EGFR-specific antibody carrying miR-16 mimics for a cancer-targeted delivery (Ref. 143). A Phase 1 clinical trial of TargomiR initiated in 2014 demonstrated the safety of the approach in 26 patients. Of the 22 patients who were assessed for response, one showed a partial response and 15 had stable disease (Ref. 144).

Tumour suppressor miR-34a downregulates the expression of oncogenes including MET, MYC, PDGFR-*α*, CDK4/6 and BCL2 (Ref. 145). Both *in vitro* and *in vivo* studies have reported that miR-34a mimics can reduce tumour growth, migration and invasion and metastasis (Ref. 146). Anti-tumour activity of co-injecting let-7 and miR-34 was demonstrated in multiple NSCLC cell lines (Ref. 147). The anti-tumour activity of this combinatorial therapy was also tested in *Kras^LSD−G12D/+^; p53^flx, flx^* mouse model of NSCLC (Ref. 147). A second *in vivo* study was then initiated using lipid-based delivery agent (NOV340) to deliver miR-34a mimics (Ref. 147). A Phase I clinical trial with MRX34, a liposomal formulation of a synthetic, double-stranded miR-34a mimic, was initiated for patients with HCC and unresectable liver metastasis. (Refs 147, 148). Unfortunately, adverse immune-mediated toxicities precluded the trial advancing to phase II (Clinical Trial identifiers: NCT01829971, NCT02862145) (Ref. 148).

Tumour suppressor miR-193a-3p is downregulated in a range of cancers including HCC (Ref. 149), NSCLC (Ref. 150) and TNBC (Ref. 151). The repression of miR-193a-3p in these cancers decreases apoptosis, increases cell proliferation and migration tumour growth and metastasis (Ref. 152). Targets of miR-193a-3p play an important role in malignant cell behaviour including KRAS (Ref. 153), ERBB (Ref. 154), and S6K2 (Ref. 155) in lung cancer, PLAU in bladder cancer (Ref. 156), MCL-1 in glioma (Ref. 157), CCND1 in prostate cancer (Ref. 158), RAB27B in osteosarcoma (Ref. 159) and SRSF2 in HCC (Ref. 160). Telford *et al*. reported that miR-193a-3p mimics reduce cancer cell proliferation/survival by inducing cell cycle arrest, apoptosis, increased cell senescence, DNA damage and inhibit migration (Ref. 161). INT-1B3, a 193a-3p mimic replacement drug, consists of a lipid nanoparticle-based delivery system (Ref. 152). In preclinical studies with tumour bearing mice, systemic injection of INT-1B3 shows significant anti-tumour activity (Ref. 152). A phase 1/1b clinical trial to investigate the safety, preliminary efficacy, pharmacokinetics and pharmacodynamics of INT-1B3 is currently ongoing (Clinical Trial identifier: NCT04675996) (Ref. 152).

### Clinical miRNA inhibition

Oncogenic miR-221-222 cluster located on the X chromosome is highly expressed in several solid tumours such as lung cancer (Ref. 162), breast cancer (Ref. 163), HCC (Ref. 164), and glioblastoma (Ref. 165) as well as haematological malignancies including myeloma (Ref. 166). In advanced cancers, upregulation of miR-221 interferes with the expression of its targets p27, p57, PUMA and PTEN promoting tumour growth (Ref. 152). Di Martino *et al*. reported anticancer effects of anti-miR221-targeted LNAs both *in vitro* and *in vivo*. LNA-i-miR-221 is a 13-mer antisense oligonucleotide that uses LNA technology and phosphorothioate backbone chemistry for increased affinity for miR221 targeting (Ref. 166). A phase I clinical trial of LNA-i-miR-221 will administer the drug via an intravenous injection to patients with multiple myeloma and advanced solid tumour (Clinical Trial identifier: NCT04811898).

Oncogene miR-155 regulates immune cell function and its overexpression affects multiple genes associated with the promotion of solid tumours including breast cancer, lung cancer, liver cancer as well as haematological malignancies including leukaemia (Ref. 167). Upregulation of miR-155 has been linked to JAK/STST, NK-KB and PI3K/AKT survival pathways stimulating T-cell receptors (Ref. 168). MiR-155 inhibition reduces proliferation and increases apoptosis in T-cell lymphoma cell lines (Ref. 168). In xenografts of B-cell lymphoma, miR-155 silencing with a LNA delivered systemically, reduced tumour burden and metastasis (Ref. 169). In another preclinical study, an anti-miR-155 molecule with a peptide nucleic acid backbone was used for greater sensitivity and efficient delivery to treat haematological malignancies (Ref. 170). These preclinical studies lead to the development of Cobomarsen which is a single-stranded, chemically modified miR-155-targeting molecule with chemical modifications for increased stability (Ref. 168). A Phase 1 trial of Cobomarsen in patients with cutaneous T-cell lymphoma (CTCL) [mycosis fungoides (MF) subtype] was initiated in 2016 and reported some therapeutic benefit for 95% of all enrolled patients with increased benefits reported for subjects who underwent more than one cycle (Ref. 168). Encouraging early data was followed by phase 2 clinical trial which started in 2019 but was terminated in 2020 due to commercial reasons (Clinical Trial identifiers: NCT03603431, NCT03713320, NCT03837457).

## Challenges for miRNA-based cancer therapies

There are several challenges in using mi-RNA based therapies including insufficient delivery to the target tissue (cancer), stability in the biological system, immune responses and unwanted off-targeting. Arguably the primary challenge for miRNA-based cancer therapies is their efficient delivery to target tissues. Tumours can have poor blood perfusion and the complexity of ECM often hinder the delivery of miRNA-based therapies. In addition, scavenging cells like TAMs, neutrophils and monocytes can prevent the miRNA carrying vehicle from reaching cancer cells (Ref. 171). To overcome these challenges, different modes of delivery are being investigated, including both viral and non-viral vector systems (Refs 172, 173). The more common viral vectors are derived from adeno-associated viruses, adenoviruses and lentiviruses, while non-viral vectors can include exosomes, polymers and liposomes (Refs 172, 173). Viral vectors have a high efficiency and have been successfully used in several clinical trials. They also form the basis of a number of FDA approved therapies. (Ref. 174). Non-viral approaches may also be beneficial. The biocompatibility and biodegradability of polymers and liposomes are some of their advantages (Ref. 175). The conjugate vehicles have selective targeting and high stability due to the use of lipid or receptor binding molecules (Ref. 176). Naturally occurring exosomes are an advantage due to their immune compatibility (Ref. 177). Both viral and non-viral systems have inherent advantages and shortcomings, therefore the choice of the delivery system should be based on the overall design of a study.

Once delivered to the target tissue, the effect of miRNA therapeutics in non-cancer cells needs to be prevented. Similarly, effects on off-target mRNAs within cancer cells is another concern as miRNAs can target multiple transcripts simultaneously (Ref. 178). MiRNA imperfect complementarity to the targeted mRNA 3′UTR has the capacity to indiscriminately silence off-target genes (Ref. 179). Another cause of off-targeting is the possibility of artificial exogenous miRNA competing with endogenous miRNA creating a dysregulation in gene expression (Ref. 179). Off-target effects can induce the silencing of tumour suppressors or activation of oncogenes in normal cells (Ref. 180). For instance, miR-15/16 cluster regulate a large proportion of the whole transcriptome in leukaemia cells (Ref. 181). Thus, these miRNAs would not likely be used as therapeutic targets. The use of cancer targeted viral and non-viral delivery vectors can also reduce unwanted off-target effects (Refs 173, 182). Furthermore, cancer cell-specific regulatory elements like tumour specific promoters can be used in the delivery system (Ref. 183). Vigilant bioinformatic and wet-lab studies need to be performed with proposed inhibitors or mimics to identify any potential off-target effects in pre-clinical studies.

Both the miRNA and the delivery vector can elicit an immune response (Ref. 184). MiRNA duplexes can trigger toll like receptor response (Refs 171, 184) leading to an interferon response against miRNA therapeutics (Ref. 171). MiRNA therapeutics designed with certain chemical modifications can mitigate these immune responses (Ref. 171). Chemical modifications can enhance the miRNA stability *in vivo*. For instance, miRNAs without chemical modification of the ribose 2′-OH are prone to nuclease-mediated degradation and have a short half-life when injected systemically. Three generations of ASOs modification techniques developed (Ref. 1) First-generation modifications substitute phosphodiester backbone with phosphothiorate to increase *in vivo* stability; (Ref. 2) Second-generation modifications substitute the 2′-O-alkyl group of the sugar moieties with 2′-OMe, 2′-O-methoxyethyl (2′MOE) or 2′-Fluoro to enhance efficacy and bioavailability and to reduce the immune stimulation and toxicity; (Ref. 3) Third-generation modifications are the chemical alteration to the furanose ring with 2′4′-methylene producing LNAs to reduce nuclease degradation and increase membrane penetration (Ref. 126).

## Concluding remarks

The inability of current therapies to effectively treat advanced or metastatic cancers stems from an incomplete understanding of the molecular mechanisms governing metastasis. Understanding molecular drivers of cancer metastasis can provide opportunities to develop novel therapeutic approaches. MiRNAs play a central role in regulating gene expression, thus, the dysregulation of miRNAs in metastasising cells warrants special attention. The dysregulation of several miRNAs is observed at every step of the metastatic process and restoring their levels is an attractive therapeutic avenue. Depending on the cancer type, dysregulated miRNAs can function as an oncogene or tumour suppressor. These dysregulations can lead to significant alterations in the expression of downstream target genes. Therapeutic approaches that utilise miRNAs aim to restore the normal levels of dysregulated miRNAs using miRNA mimics and inhibitors. There are several studies which report a successful use of miRNA mimics and inhibitors in pre-clinical *in vitro* and *in vivo* studies for targeting both primary tumours and metastasis for different cancer types. This has led to the initiation of human clinical trials using miRNA-based therapies for both solid and blood cancers. However, there are both technical and practical limitations for delivering miRNA-based therapies to patients. The *in vivo* stability and delivery of these miRNAs at therapeutic levels to the target tissue is a major issue for clinical applications. Similarly, therapy-induced toxicity and potential off-target effects are major concerns. There are several ongoing developments in this area to increase the stability of miRNAs mostly involving chemical modifications. Similarly, developments in the field of both viral and non-viral vector-based delivery can make the therapy cancer-specific and reduce off-target effects. Further research needs to be performed in order to identify novel miRNAs which control metastasis and potential therapeutic target; only then will the full potential of miRNA-based therapies for cancer metastasis be realised.

Abbreviations: BRF2, TFIIB-related factor 2, CADM1, Cell Adhesion Molecule 1, CDH1, Cadherin-1 or Epithelial cadherin, CDH5, Cadherin 5 or VE-Cadherin, CSF1, Colony-stimulating factor-1, EIF5A2, Eukaryotic Translation Initiation Factor 5A2, FAK, Focal adhesion kinase, HMGA2, High-mobility group AT-hook 2, HOXA10, Homeobox A10, HOXD10, Homeobox D10, IRF2, Interferon regulatory factor 2, IL-10, Interleukin 10, KLF4, Krüppel-like factor 4, KRAS, Kirsten rat sarcoma viral oncogene homologue, MMP2, Matrix metalloproteinase-2, MYC, MYC proto-oncogene, bHLH transcription factor, NANOG, Nanog Homeobox, N-RAS, Neuroblastoma RAS viral oncogene homologue, Oct-4, Octamer-binding transcription factor 4, PDCD4, Programmed Cell Death 4, PTEN, Phosphatase and tensin homologue, PTPN9, Tyrosine-protein phosphatase non-receptor type 9, PTPRU, Protein Tyrosine Phosphatase Receptor Type U, ROCK1, Rho Associated Coiled-Coil Containing Protein Kinase 1, SOX2, SRY-Box Transcription Factor 2, SOX4, SRY-Box Transcription Factor 4, TGF-*β*, Transforming growth factor beta, TGFBR3, Transforming growth factor beta receptor 3, VEGF, Vascular Endothelial Growth Factor, WIF1, WNT Inhibitory Factor 1, Wnt5a, Wnt Family Member 5A, ZEB1, Zinc finger E-box binding homeobox 1, ZEB2, Zinc finger E-box-binding homeobox 2, ZO-1, Zonula occludens-1. Cancers: Breast, Colon, CRC, Colorectal cancer, Gastric, HCC, Hepatocellular carcinoma, NSCLC, non-small cell lung cancer, pancreatic, PDAC, Pancreatic ductal adenocarcinoma, uveal melanoma.
